# Analyses of eye movement parameters in children with anisometropic amblyopia

**DOI:** 10.1186/s12886-024-03539-x

**Published:** 2024-07-09

**Authors:** Yunwei Fan, Huaxin Zuo, Ping Chu, Qian Wu, Li Li, Yuan Wang, Wenhong Cao, Yunyu Zhou, Lijuan Huang, Ningdong Li

**Affiliations:** 1 Department of Ophthalmology, Beijing Children’s Hospital, Capital Medical University, National Center for Children’s Health, Beijing, 100045 China; 2 Beijing Key Laboratory for Pediatric Diseases of Otolaryngology, Head and Neck Surgery, Beijing Children’s Hospital, Beijing Pediatric Research Institute, Capital Medical University, National Center for Children’s Health, Beijing, 100045 China; 3 The second affiliated hospital of Fujian medical university, Fujian, 362000 Zhejiang China

**Keywords:** Eye movement, Anisometropic amblyopia, Saccade, Accommodation, Children

## Abstract

**Objective:**

To investigate the characteristics of eye movement in children with anisometropic amblyopia, and to compare those characteristics with eye movement in a control group.

**Methods:**

31 children in the anisometropic amblyopia group (31 amblyopic eyes in group A, 31 contralateral eyes in group B) and 24 children in the control group (48 eyes in group C). Group A was subdivided into groups Aa (severe amblyopia) and Ab (mild-moderate amblyopia). The overall age range was 6–12 years (mean, 7.83 ± 1.79 years). All children underwent ophthalmic examinations; eye movement parameters including saccade latency and amplitude were evaluated using an Eyelink1000 eye tracker. Data Viewer and MATLAB software were used for data analysis.

**Results:**

Mean and maximum saccade latencies, as well as mean and maximum saccade amplitudes, were significantly greater in group A than in groups B and C before and after treatment (*P* < 0.05). Mean and maximum saccade latencies were significantly different among groups Aa, Ab, and C (*P* < 0.05). Pupil trajectories in two detection modes suggested that binocular fixation was better than monocular fixation.

**Conclusions:**

Eye movement parameters significantly differed between contralateral normal eyes and control eyes. Clinical evaluation of children with anisometropic amblyopia should not focus only on static visual acuity, but also on the assessment of eye movement.

## Introduction

Amblyopia is a common developmental disorder of vision in children, involving abnormal binocular reciprocal inhibition or form deprivation without any organic cause; the disorder affects monocular or binocular spatial visual function [1], and its prevalence is 1–6% [1]. Amblyopia is characterized by abnormal vision, as well as abnormal eye movements related to vision [2].

Eye movements include saccades, smooth tracking, optokinetic nystagmus (OKN), and vestibulo-ocular reflex (VOR). Whereas the VOR is not directly related to vision—it functions through the influence of vestibular function on eye movement—the other three eye movements are closely related to vision. Smooth tracking depends on capture by the macula and requires the involvement of vestibular function. OKN primarily reflects peripheral retina function, which is a compensatory mechanism. Saccade is an innate ability that mainly reflects central retina function, involving eye-mediated capture of moving objects, information processing by the brain, and a rapid systemic response. Accordingly, saccade movement directly reflects visual function and eye-brain-body coordination.

Amblyopia is considered to be associated with abnormalities in the brain cortex, which is one of the crucial organs involved in visual attention and the generation of eye movements such as saccades and smooth pursuits. [3]. To accurately evaluate central vision status, body and head positions must be stable during examinations of eye movement. The current criteria for diagnosis and successful treatment of amblyopia are assessed via monocular vision examination [4]. Although normal visual acuity can be achieved after amblyopia treatment, previous studies have shown that numerous patients continue to exhibit lower contrast sensitivity visual acuity, compared with individuals who have normal vision.

Here, we prospectively analyzed binocular visual function in 31 children with anisometropic amblyopia and 24 children with normal vision who attended the outpatient department of Beijing Children’s Hospital. We investigated characteristic changes in dynamic vision (i.e., saccades) in children with anisometropic amblyopia. We aimed to identify relationships between saccade parameters and visual impairment severity. Whether differences remain in the saccadic function of the “original amblyopic eye” compared to the normal eye when the visual acuity reaches the criteria for cured vision in patients with amblyopia was investigated in this study.

## Methods

### Study design and participants

This study is a cross-sectional case-control study, and the data were collected from children who attended the Department of Ophthalmology at Beijing Children’s Hospital from October 2020 to January 2022. Children aged 6–12 years (mean 7.83 ± 1.79 years) with anisometropic amblyopia (31 participants, 62 eyes) and children with normal vision (24 participants, 48 eyes) were included. 31 children with anisometropic amblyopia were divided into a group of amblyopic eyes (31 eyes in group A) and a group of contralateral eyes (31 eyes in group B). According to amblyopia severity, group A was divided into groups Aa (severe amblyopia with visual acuity < 20/100) and group Ab (mild-moderate amblyopia with visual acuity ≥ 20/100) [1, 5]. In Group A, there were 29 children with hyperopic spherical equivalent (4 cases ≤ + 3.00 diopter sphere (DS), 19 cases between > + 3.00 DS and ≤ + 6.00 DS, and 6 cases with > + 6.00 DS), and 2 children with myopic spherical equivalent, both less than − 3.00 DS. Children with normal vision (24 participants, 48 eyes) were included in the control group (group C).

All children with amblyopia received the same amblyopia treatment, and changes were observed 6–10 months after treatment. The following treatments were provided: eyes in group B were covered for 4–6 h daily [5]; the contralateral eyes were covered for 6 h in the severe amblyopia group (group Aa) and 4 h in the mild-moderate amblyopia group (group Ab). Eyes in group A received binocular visual stimulation and perceptual training for 30 min daily; all eyes in children with anisometropic amblyopia received simultaneous visual perception training, which was performed outside the 4–6-hour monocular occlusion period.

### Inclusion and exclusion criteria

To identify anisometropic amblyopia, refractive error correction was performed after retinoscopy with ophthalmic gel (1% atropine sulfate); clinical diagnoses of amblyopia were made after children had worn corrective lenses for 3 months [4, 5]. Based on the developmental pattern of visual acuity in children, the normal lower limit for visual acuity is 20/40 for children aged 3–5 years and 20/25 for children aged 6 years and above. Visual acuity lower than these standards is considered diagnostic of amblyopia [5, 6]. Each child met at least one of the following inclusion criteria: binocular spherical degree difference ≥ 1.50 spherical diopter (D), binocular cylindrical degree difference ≥ 1.00 cylindrical D, or binocular visual acuity difference ≥ 2 lines [4, 6].

Normal vision (i.e., control eyes) was regarded as normal binocular vision and spherical equivalent of + 0.50 D to -0.25 D [7].

Participants were excluded if they met any of the following criteria: lack of foveal fixation; absence of normal development and/or presence of neurological or other systemic diseases; presence of strabismus (except heterophoria); presence of conjunctivitis, dry eye, cataract, glaucoma, ptosis, fundus disease, or any ocular disorder; and lack of cooperation during the examination and/or failure to complete the 6 to10-month course of treatment.

### Assessment

The initial examination comprised retinoscopy after mydriasis with ophthalmic gel (1% atropine sulfate), correction of distance visual acuity to international standards, external eye movement, eye alignment assessment, slit lamp evaluation, and fundus photography.

Eye movement assessment: all examinations were conducted by a technician using an Eyelink 1000 eye tracker [8]. Refractive error correction was performed for eyes with anisometropic amblyopia. All participants underwent eye movement assessment of the right eye, left eye, and both eyes separately. During monocular examination, the other eye was covered with an eye patch. Calibration and verification were conducted before formal detection of eye movement [9]; specifically, the pupillary imaging position was consistent with the target in the region of interest on the screen. Four boundary points were selected within the region of interest. Horizontal and vertical stimuli were repeated eight times, with a duration of 2 s per fixation and a sampling rate of 1000 Hz.

### Data analysis

**Fig. 1:**
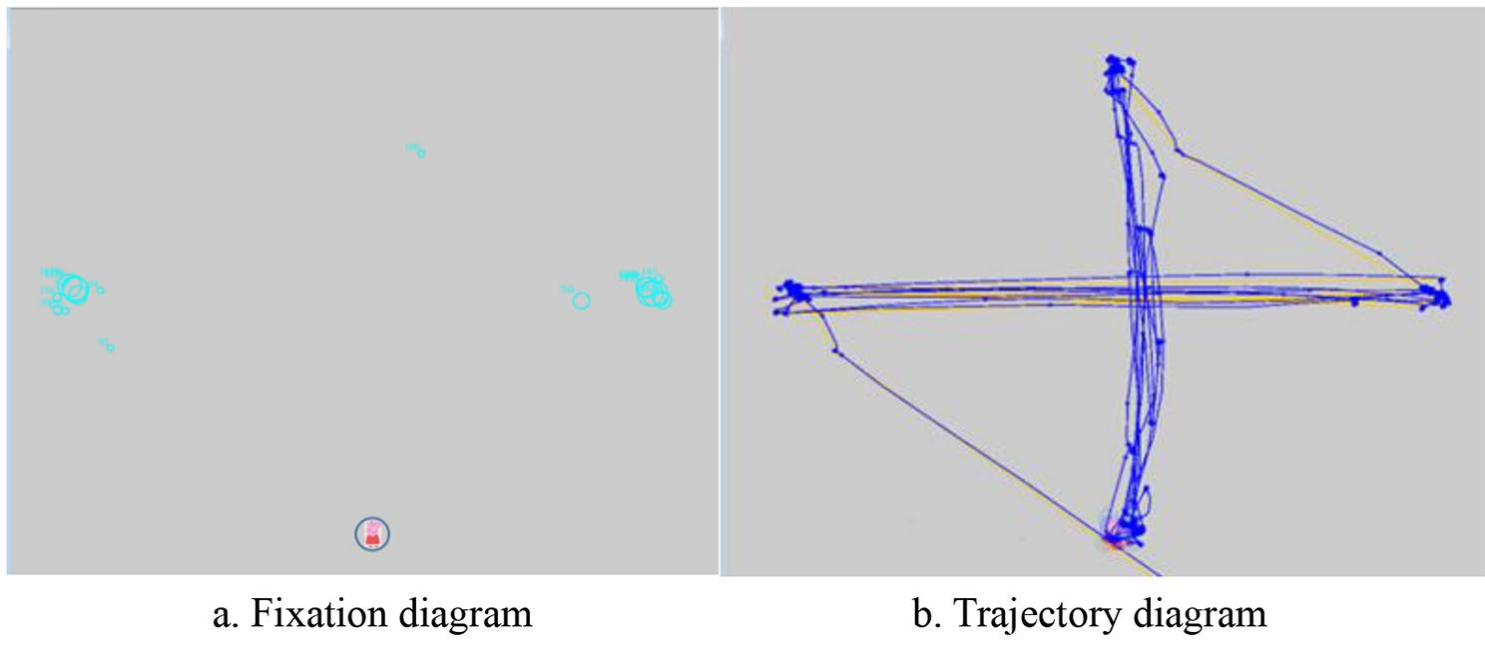
Pupil fixation position and trajectory. (**a**). Greater overlap of two horizontal green circles indicates better fixation. (**b**). Pupil trajectory was captured during target tracking. Both parameters have been positively correlated with good visual function

Data Viewer and MATLAB software were used for data analysis. Data Viewer was used to organize data and generate figures (Fig. 1), including pupillary fixation position, pupil trajectory, and coordinate diagrams corresponding to the pupil trajectories in both eyes. Fixation position and trajectory data were used for rapid assessment and analysis during examination and diagnosis.

**Fig. 2:**
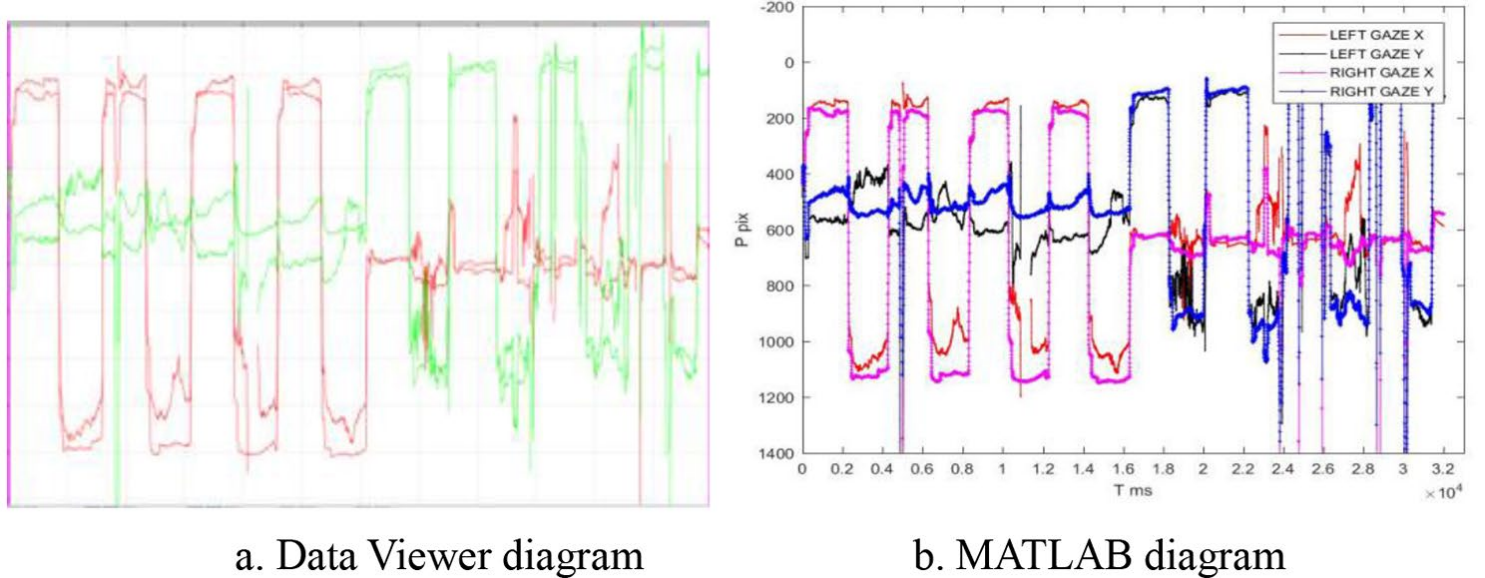
Coordinate diagrams of pupil trajectory. (**a**). Coordinate diagram of binocular pupil position in children with anisometropic amblyopia displayed by Data Viewer, which can analyze interactions and differences between eyes with respect to vision. (**b**). Coordinate diagram of pupil position in both eyes determined using relevant data from MATLAB self-programmed report, similar to the results shown in Fig. 2a

MATLAB was used to analyze the extracted data. Based on previous studies and clinical requirements, six potentially effective parameters were analyzed [10]: saccade latency (mean and maximum), saccade amplitude (mean, maximum, and minimum), and microsaccade amplitude (Fig. 2).

### Statistical analyses

Statistical analyses were performed with SPSS software Version 22.0. Data were described as means ± standard deviations (SDs). The Shapiro-Wilk method was used to exam the normal distribution of the data. Categorical data were analyzed using the chi-squared test. Analysis of variance was used to compare normally distributed measurement data among three groups. P-values < 0.05 were considered statistically significant.

## Results

### Comparison of pre-treatment eye movement parameters among groups

All data are normally distributed. Six key parameters of eye movement (e.g., saccade latency, saccade amplitude, and microsaccade amplitude) were compared among the three groups before treatment (Table 1). The results showed significant differences in the mean and maximum saccade latencies, as well as the mean and maximum saccade amplitudes (*P* < 0.05). The mean and maximum saccade latencies were significantly longer in group A than in the other two groups (*P* < 0.05). The mean and maximum saccade latencies and the mean and maximum saccade amplitudes were significantly greater in group B than in group C (*P* < 0.05).

**Table 1:**
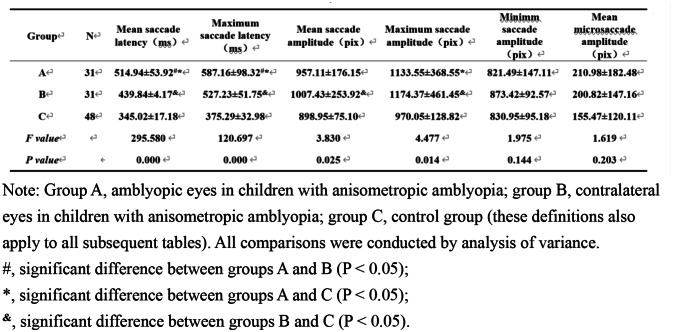
Analysis of variance (ANOVA) and posthoc comparisons of pre-treatment eye movement parameters among groups (mean ± SD)

Group A was divided into two groups according to amblyopia severity; groups Aa, Ab, and C were compared (Table 2). The results showed that the mean and maximum saccade latencies, as well as the maximum saccade amplitude, were significantly different among the three groups (*P* < 0.05). Notably, latency was significantly longer in group Aa than in the other two groups (*P* < 0.05).

**Table 2:**
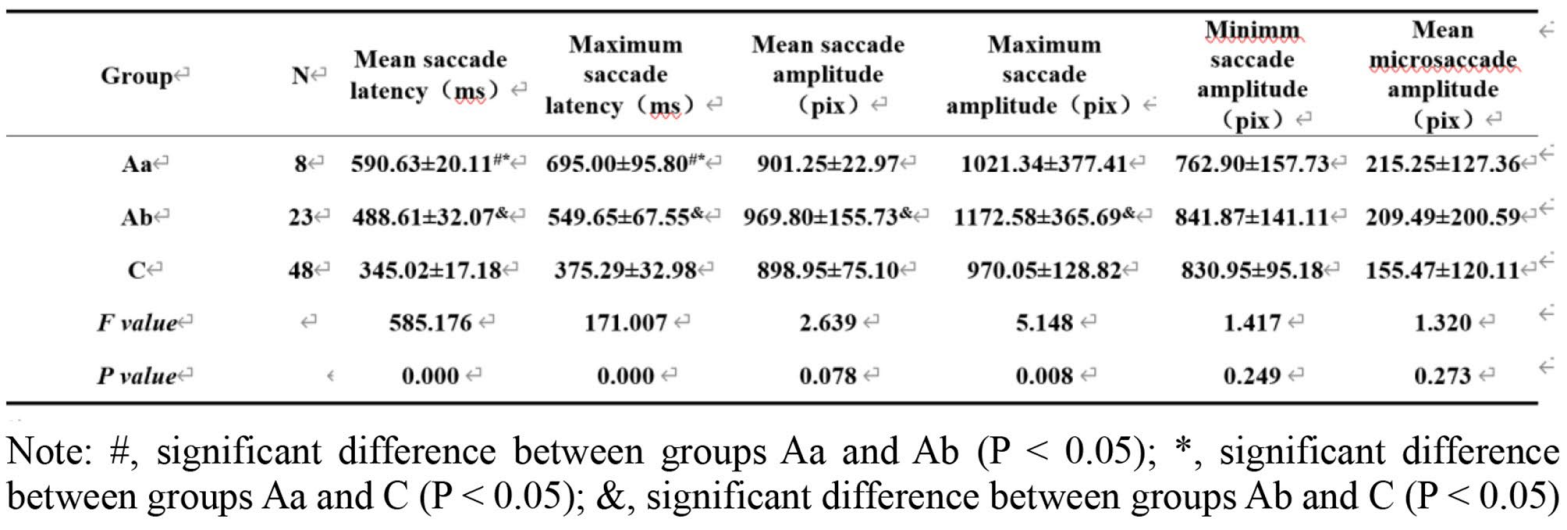
Comparison of pre-treatment eye movement parameters between groups with different amblyopia severity and control group (mean ± SD)

### Eye movement trajectory and square-wave pattern in the control and anisometropic amblyopia groups were analyzed during pre-treatment monocular and binocular fixation, respectively

**Fig. 3:**
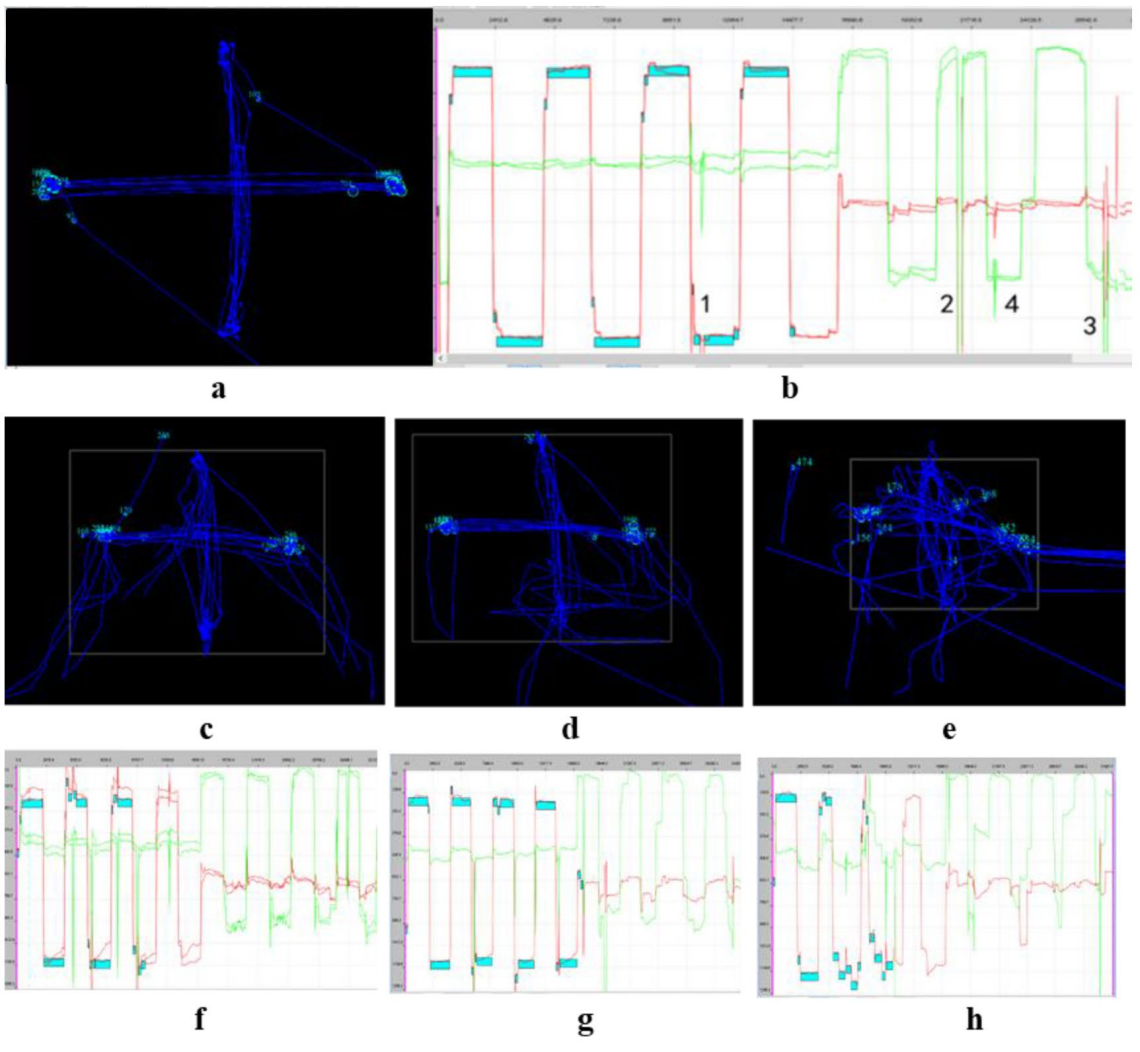
Eye movement trajectory (**a**) and square-wave pattern of pupil fixation position (**b**) in the control group during binocular detection. Analysis of eye movement detection in an anisometropic amblyopia case (corrected visual acuity: right eye 20/25, left eye 20/80): c-e depict respective eye movement trajectories during detection in both eyes, right eye, and left eye; f–h show respective square-wave patterns of pupil fixation position during detection in both eyes, right eye, and left eye

Eye movement characteristics in various visual acuity conditions significantly differed between the two groups, regardless of external factors (e.g., lack of cooperation) (Fig. 3).

As shown in Fig. 3c–e, although the trajectories are generally chaotic, the trajectory of binocular fixation is better than the trajectory of monocular fixation. During left eye fixation, the pupil position substantially deviates from the target (Fig. 3e); the deviation is smaller during binocular fixation and right eye fixation (Fig. 3c and d). The square-wave patterns in Fig. 3f-h indicate poor adjustment to horizontal targets in both monocular and binocular fixation; this mainly affects left eye fixation. Overshoot and undershoot were evident during fixation, with shorter fixation time and frequent adjustment in a single fixation stage (e.g., later stages).

### Comparison of post-treatment eye movement parameters among groups

Comparisons of six key eye movement parameters after treatment (Table 3) showed that the mean and maximum saccade latencies, as well as mean and maximum saccade amplitudes, significantly differed among the three groups (*P* < 0.05). Pairwise comparisons revealed that the mean and maximum saccade latencies were significantly longer in group A than in the other two groups (*P* < 0.05). The mean and maximum saccade latencies, as well as the mean and maximum saccade amplitudes, were significantly longer in group B than in group C (*P* < 0.05).

**Table 3:**
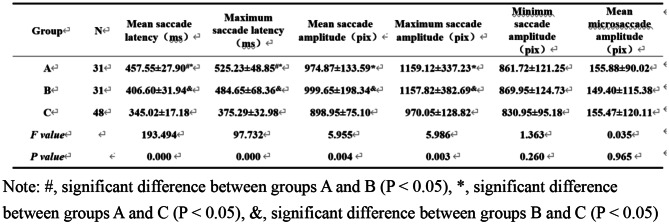
Comparison of post-treatment eye movement parameters among groups (mean ± SD)

### Comparison of pre- and post-treatment eye movement parameters between groups a and C

Comparisons of eye movement parameters between group A before and after treatment and group C showed significant differences in mean and maximum saccade latencies, as well as maximum saccade amplitude (Table 4). Pairwise comparisons among the three groups revealed that the mean and maximum saccade latencies after treatment were significantly different from those parameters before treatment, suggesting that saccade latency is significantly improved after treatment, but it remains significantly different from the findings in group C (*P* < 0.05).

**Table 4:**
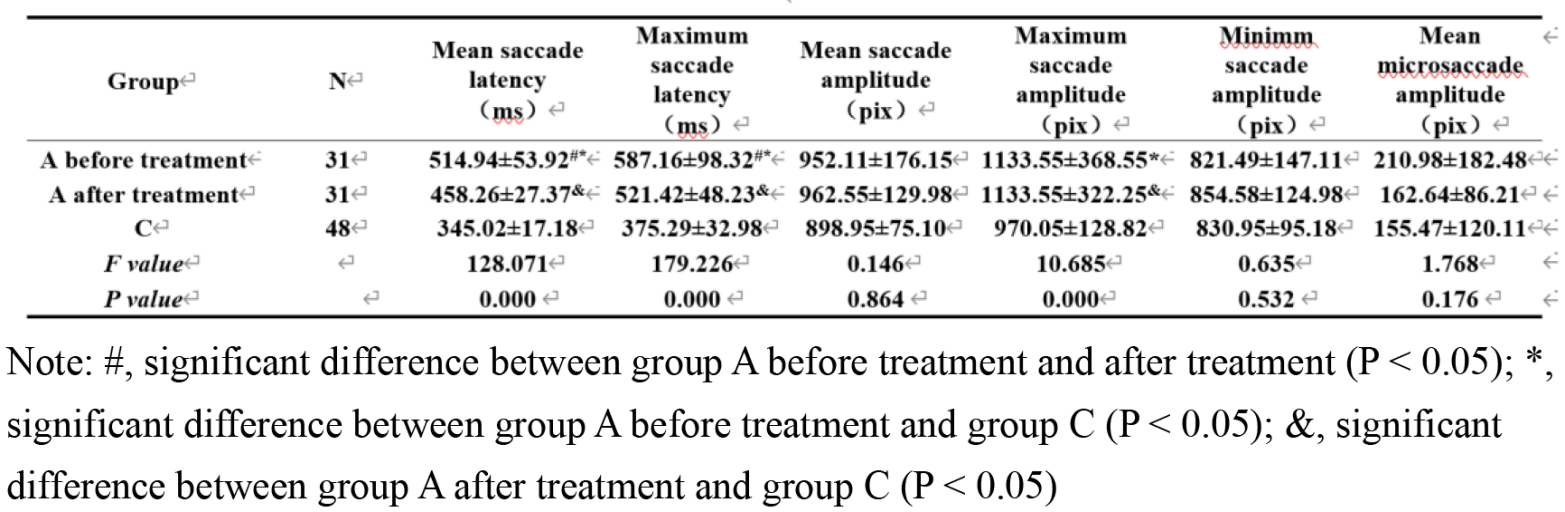
Comparison of pre- and post-treatment eye movement parameters between groups A and C (mean ± SD)

## Discussion

Amblyopia is a developmental disorder of the central nervous system caused by abnormal processing of visual images [6], often accompanied by the loss of various sensory functions, as well as structural and functional abnormalities that affect visual pathways [11, 12]. In anisometropic amblyopia, interocular competition occurs, such that the weaker eye is suppressed [13]. During sensitive periods of visual development, long-term suppression can lead to loss of retinal input and a decline in regulatory function [14, 15]. Additionally, anisometropia disrupts binocular vision with increasing severity as the degree of anisometropia increases [16]. Recent studies regarding children with anisometropic amblyopia have focused on diagnoses that involve static vision. Children with anisometropic amblyopia display reduced visual acuity in one eye, as well as abnormalities in contrast sensitivity and regulatory function in both amblyopic and contralateral eyes [17]. However, characteristic changes in eye movement among children with anisometropic amblyopia have not been thoroughly investigated; thus, such changes remain controversial and are rarely used for clinical assessment.

Here, we attempted to eliminate most confounding factors, select children with typical anisometropic amblyopia, and conduct in-depth research regarding their eye movement. This approach supports the analysis of differences between amblyopic and contralateral normal eyes in individual children, as well as differences between the amblyopia and control groups. Furthermore, it can help to clarify relationships between eye movement parameters and vision, while establishing the importance of a comprehensive diagnosis of amblyopia.

### Analysis of eye movement characteristics in amblyopic, contralateral, and control eyes

Key pre- and post-treatment eye movement parameters in 31 children with anisometropic amblyopia were subjected to pairwise comparison. The results showed that four parameters (mean saccade latency, maximum saccade latency, mean saccade amplitude, and maximum saccade amplitude) were significantly different among groups A, B, and C. Latencies were significantly longer in group A than in the other two groups; amplitudes were significantly greater in group A than in group C. The results of previous studies have suggested that the neural centers involved in saccades constitute superior and inferior centers [11, 18]. Damage to the central conduction pathway, particularly the visual cortex, affects saccades. Saccade latency mainly reflects the central conduction time, whereas saccade amplitude reflects positioning and fixation ability. Amblyopic eyes are deficient in movement and positioning. Regardless of positioning accuracy, repositioning is necessary after saccade correction (through overshoot or undershoot). Previous literature regarding cortical damage in amblyopic children [16] is consistent with a prolonged conduction time that results in increased saccade latency; poor eyesight affects positioning ability, leading to frequent adjustment of the amblyopic eye and increased saccade amplitude. Additionally, saccade latency was significantly longer in children with severe amblyopia than in the other two groups. Furthermore, children with severe amblyopia exhibit obvious defects in eye movement and positioning, along with poor fixation, coordination, and positioning. Thus, their saccadic pupil trajectory and square-wave pattern indicated target positioning accuracy that required correction by multiple instances of overshoot and undershoot, which may also reflect positioning defects induced by damage to the visual cortex.

### Amblyopic eyes display synergistic interactions with contralateral eyes

This comprehensive study of anisometropic amblyopia revealed key eye movement parameters in children with amblyopia; the findings also suggested that the amblyopic and contralateral eyes suppress and complement each other. Notably, 90% of individuals with normal vision have binocular vision that is ≥ 40% better than monocular vision [19]. Binocular vision damage causes binocular vision to be worse than monocular vision [20]. Amblyopic eyes exhibit synergistic enhancement involving contralateral eyes. Among children with normal vision and amblyopia, visual function is better in binocular fixation than in monocular fixation.

In this study, the eye movement parameters of children with anisometropic amblyopia and children in the control group were recorded during fixation and tracking. The eye movement results in various visual acuity conditions significantly differed between the two groups, regardless of external factors such as lack of cooperation. Examination and calibration both were uncomplicated in the control group. The results showed that fixation was prolonged and stable, with small fluctuations in x and y values, less eyelid twitching during fixation, fast tracking speed, high accuracy, and robust consistency in binocular parameters. However, eye movement calibration was difficult to conduct in children with severe amblyopia, and an auxiliary device was required. Based on eye movement trajectory, we suspected that eye tracking could reflect the degree of amblyopia severity. Poor visual acuity hinders eye tracking, emphasizes fixation instability, and produces suboptimal results. Eye movement reflects the ability of the visual cortex to process visual information. In a functional magnetic resonance imaging study, Wen et al. [21] found that the functions of the lateral geniculate body–V1 small cell pathway and the cortex–superior colliculus pathway were abnormal in adults with amblyopia. The reduced response of the amblyopic eye and enhanced response of the contralateral eye indicated that the superior colliculus was more dependent on visual information from the contralateral eye to control eye movement and attention. These findings are consistent with the understanding of the auxiliary compensation effect of the contralateral eye on the amblyopic eye in patients with anisometropic amblyopia [21].

Moreover, previous research showed that microsaccades reflect visual acuity of 0–0.6° at the center of the macula. Worse visual acuity leads to increases in eye drift amplitude, microsaccade amplitude, and amplitude variability, all of which are positively correlated. Microsaccades induce neuronal regulation in the cortical area V1 and extrastriate cortex, with important roles in concealing neural adaptation and preventing visual decline [22, 23]. Additionally, vision is improved after temporary suppression of visual sensation related to microsaccades. In the present study, we observed frequent occurrences of saccades, microsaccades, significant undershoot/overshoot, and large amplitude fluctuations in amblyopic eyes. These findings suggest a decrease in neural activity suppression within the superior colliculus of amblyopic eyes. However, adjustment continues via saccades and microsaccades, rather than “laziness,” to compensate for visual defects.

### Contralateral healthy eyes are not identical to normal control eyes

Amblyopia is positively correlated with the degree of fixation instability, but normal contralateral eyes in children with anisometropic amblyopia also exhibit fixation instability compared with control eyes. This phenomenon is regarded as “initial signal abnormalities” in contralateral eyes [16]. Here, we compared the contralateral eyes of children with anisometropic amblyopia (group B) to control eyes (group C); we found that both latency and amplitude were greater in group B than in group C before and after treatment. We also found that the contralateral eyes of children with anisometropic amblyopia exhibited function that differed from normal eye function; these eyes were suppressed by amblyopia (i.e., they experienced changes involving V5 in the visual conduction pathway), resulting in abnormal saccades in monocular and binocular fixation. The phenomenon was most prominent in monocular fixation. Therefore, in the treatment of anisometropic amblyopia, although contralateral eye vision may be normal, both eyes require varying degrees of functional treatment to promote recovery from “temporary decompensation” that occurs in the contralateral eye. Previous literature suggested that the treatment effect of patching for severe amblyopia with 6 h of daily patching, is equivalent to full-day patching, while mild amblyopia may only require 2 h of patching [1]. Since the severity of amblyopia varies among our participants, to standardize the treatment protocol, occlusion time was set at 4–6 h for all children. This ensured maximum stimulation for the amblyopic eye and repairment of the cerebral cortex.

### Function slightly differs between treated amblyopic eyes and normal control eyes

The analysis of six key parameters in group A before and after treatment revealed that only the mean and maximum saccade latencies were significantly different in pairwise comparisons; those latencies were significantly lower after treatment than before treatment. A possible explanation is that after amblyopia treatment, the brain demonstrates greater ability to participate in visual attention and eye movement, resulting in a shortened latency period. Additionally, eye movement primarily reflects the corresponding brain function and positioning necessary to process visual information. Saccadic amplitude represents the autonomic movement of the eyes and spatially shifting process of the gaze center, reflecting positioning function capacity. Thus, only an improvement in saccade positioning ability can lead to a reduced amplitude or smaller fluctuations. The abnormal binocular visual function in anisometropic amblyopia may be related to defects in the visual cortex [16]. In the present study, we found saccadic amplitudes of some participants fluctuated greatly before and after treatment, but no significant differences. This preliminary observation suggests that although visual acuity may improve, the short duration of brain visual cortex repairment and the inability to accurately establish visual function may be related. Thus, a longer treatment time is needed to observe changes in eye movement parameters and their underlying mechanisms of action.

## Conclusion

Overall, anisometropic amblyopia in children affects visual acuity, damages binocular vision integrity, and directly influences visual quality. Through our study on visual acuity and eye movement parameters, the correlation between amblyopia and eye movement was obtained. Our findings indicated that in addition to static vision, the dynamic vision of anisometropic amblyopia is also impacted, and may become an important part of future clinical evaluations. However, this study was limited by factors such as age and cognitive abilities. There was a shortage of samples for groups of younger age and severe amblyopia, and the clinical observation time was relatively short. Additionally, though results of the pairwise comparison of the three groups have shown the significant difference of eye movement parameters between groups before and after treatment, multiple comparisons were not performed in this study, which will be add in future research. In the prospective cohort study that we are currently conducting, the grouping based on different age, severity of amblyopia, and treatment durations are refined. Parameters are selected rationally to conduct a more precise correlation analysis between visual acuity and eye movement parameters, aiming to provide clinical evidence for standardized diagnosis and personalized treatment of amblyopia.

## Data Availability

The datasets generated and/or analyzed during the current study are not publicly available since the following-up research including treatment and effect on these patients is still carrying on. But they are available from the corresponding author on reasonable request.
